# Editorial for Special Issue “Osteoclastogenesis and Osteogenesis: Physiological and Molecular Responses to Xenobiotics and Biomaterials”

**DOI:** 10.3390/cimb48050537

**Published:** 2026-05-21

**Authors:** Maria Giovanna Rizzo

**Affiliations:** Department of Chemical, Biological, Pharmaceutical and Environmental Sciences, University of Messina, 98166 Messina, Italy; mgrizzo@unime.it

We are pleased to present this Special Issue of Current Issues in Molecular Biology, entitled “Osteoclastogenesis and Osteogenesis: Physiological and Molecular Responses to Xenobiotics and Biomaterials”. This collection groups original research articles and review papers that address the complex biological responses of bone tissue to external stimuli, including biomaterials, pharmacological agents, and physical factors. Together, these contributions provide a comprehensive overview of how external stimuli and biomaterials influence the balance between osteogenesis and osteoclastogenesis at both physiological and molecular levels.

The maintenance of bone tissue depends on a continuous and exceptionally tuned remodeling process, in which formation and resorption are closely coordinated. This balance, driven by the activity of osteoblasts and osteoclasts, is essential for not only preserving skeletal structure but also enabling adaptive responses to physiological and pathological conditions. When this equilibrium is altered, the consequences can range from defective healing to progressive bone loss, highlighting the need for a deeper understanding of the mechanisms involved [1,2,3,4].

Recent advances in bone biology and regenerative research have significantly improved our understanding of the physiological and molecular mechanisms governing skeletal remodeling and repair. Bone regeneration is a highly coordinated process involving the interplay of osteoblasts, osteoclasts, extracellular matrix components, and local microenvironmental signals, all of which contribute to maintaining skeletal homeostasis. Increasing evidence indicates that biochemical mediators, mechanical stimuli, xenobiotics, and biomaterial-derived cues can profoundly influence cellular differentiation, matrix remodeling, and osteoimmune responses through the modulation of interconnected signaling pathways. In this context, elucidating the molecular events underlying bone cell responses is essential for not only understanding physiological bone turnover but also identifying innovative therapeutic strategies aimed at enhancing tissue regeneration and restoring bone functionality under pathological conditions [5,6,7,8].

The original research articles included in this Special Issue provide important insights into the molecular mechanisms underlying osteogenic responses. One study investigates the effects of pulsating fluid shear stress on pre-osteoblasts, demonstrating that low, but not high, mechanical stimulation modulates the expression of matrix extracellular phosphoglycoprotein (MEPE) through integrin β subunits. These findings highlight the relevance of mechanotransduction pathways in regulating osteoblast function and suggest that the magnitude of mechanical stimuli plays a critical role in bone cell responses [9].

Another original contribution focuses on the osteogenic differentiation of human dental pulp stem cells (DPSCs) induced by a helioxanthin derivative. Through integrated microRNA–mRNA analyses, this study identifies complex regulatory networks involving extracellular vesicle-associated miRNAs and key osteogenic genes. The results demonstrate enhanced alkaline phosphatase activity and upregulation of osteogenic markers, including collagen type I and WNT signaling components, providing new insights into the molecular mechanisms driving stem cell differentiation and highlighting the therapeutic potential of extracellular vesicle (EV)-mediated regulation [10].

The review articles included in this Special Issue expand the discussion by addressing emerging strategies and innovative approaches in bone regeneration. A comprehensive review on extracellular vesicles describes their central role as mediators of intercellular communication, emphasizing their ability to transport bioactive molecules such as proteins, lipids, and nucleic acids. These vesicles regulate osteogenesis, angiogenesis, and immune responses, and represent promising cell-free therapeutic tools for enhancing bone repair, although challenges related to standardization and clinical translation remain [11].

Another review in this Special Issue focuses on marine-derived biomaterials as innovative and sustainable tools for bone regeneration. These materials, including calcium carbonate, silica-based compounds, polysaccharides, and bioactive peptides, exhibit unique biochemical properties that promote osteoinduction, osteoconduction, and osseointegration. Importantly, they are capable of activating key molecular pathways such as BMP/Smad and Wnt/β-catenin signaling, which are essential for osteoblast differentiation and bone matrix formation [12], with emerging evidence highlighting the use of phage display technology to identify and engineer bioactive peptides with enhanced osteogenic potential.

In addition, a systematic review evaluates the use of teriparatide in guided bone regeneration, analyzing preclinical studies across various craniofacial models. The findings consistently demonstrate that teriparatide enhances osteogenesis, angiogenesis, and graft integration, particularly when combined with biomaterials. However, the review also highlights methodological limitations in the available studies, including risks of bias and the lack of standardized protocols, underlining the need for further well-designed clinical trials [13].

Overall, the contributions collected in this Special Issue emphasize the multifactorial nature of bone remodeling and regeneration. They highlight how molecular signaling pathways, mechanical stimulation, and biomaterial properties converge to regulate osteogenic and osteoclastogenic processes. Importantly, these studies reflect a shift toward more advanced and integrative approaches in regenerative medicine, where biological, physiological, and material sciences are combined to design more effective therapeutic strategies.

We hope that this Special Issue will provide valuable insights for researchers and clinicians working in the field of bone biology and regenerative medicine and that it will stimulate further investigations aimed at improving our understanding of bone responses to external stimuli and at developing innovative solutions for clinical applications.

## References

[1] Wu Y., Gan D., Liu Z., Qiu D., Tan G., Xu Z., Xue H. (2025). Osteocytes: Master Orchestrators of Skeletal Homeostasis, Remodeling, and Osteoporosis Pathogenesis. Front. Cell Dev. Biol..

[2] Zhang K., Li H., Wang T., Li F., Xie Z., Luo H., Zhu X., Kang P., Kang Q., Fei Z. (2025). Mechanisms of Bone Regeneration Repair and Potential and Efficacy of Small Molecule Drugs. Biomed. Pharmacother..

[3] Wu Z., Li W., Jiang K., Lin Z., Qian C., Wu M., Xia Y., Li N., Zhang H., Xiao H. (2024). Regulation of Bone Homeostasis: Signaling Pathways and Therapeutic Targets. MedComm.

[4] Zhu S., Yan M.-Q., Masson A., Chen W., Li Y.-P. (2026). Cell Signaling and Transcriptional Regulation of Osteoclast Lineage Commitment, Differentiation, Bone Resorption and Diseases. Cell Discov..

[5] Sabouri Z., Dequeecker M., Anees H., Rastegar Adib F., Jamous R., Zheng J., Lyu X., Stoetzel S., Heiss C., El Khassawna T. (2025). Recent Advances in Biomaterials for Bone Regeneration: Bridging Innovation and Clinical Translation. Mater. Today Bio.

[6] Cai W., Huo Y., Liu Y., Su Y., Guo H., Wang L., Li B., Liang T. (2025). Biomechanics in Bone Regeneration and Mechanobiology in Osteoblasts: Fundamental Concepts and Recent Progress. Eng. Med..

[7] Dalle Carbonare L., Mottes M., Valenti M.T. (2025). The Bone Microenvironment: New Insights into the Role of Stem Cells and Immune Cells in Bone Regeneration. Stem Cell Res. Ther..

[8] Sanati M., Pieterman I., Levy N., Biehl J.K., Habibovic P. (2025). Osteoimmunomodulation by Bone Implant Materials: Harnessing Physicochemical Properties and Chemical Composition. Biomater. Sci..

[9] Jin J., Zandieh-Doulabi B. (2024). Low, but Not High, Pulsating Fluid Shear Stress Affects Matrix Extracellular Phosphoglycoprotein Expression, Mainly via Integrin β Subunits in Pre-Osteoblasts. Curr. Issues Mol. Biol..

[10] Fujii Y., Minami S., Hatori A., Kawase-Koga Y., Ogasawara T., Chikazu D. (2024). Integrated MicroRNA-mRNA Analyses of the Osteogenic Differentiation of Human Dental Pulp Stem Cells by a Helioxanthin Derivative. Curr. Issues Mol. Biol..

[11] Biswas S., Gangadaran P., Dhara C., Ghosh S., Phadikar S.D., Chakraborty A., Mahajan A.A., Mondal R., Chattopadhyay D., Banerjee T. (2025). Extracellular Vesicles in Osteogenesis: A Comprehensive Review of Mechanisms and Therapeutic Potential for Bone Regeneration. Curr. Issues Mol. Biol..

[12] Rizzo M.G., Briglia M., Zammuto V., Morganti D., Faggio C., Impellitteri F., Multisanti C.R., Graziano A.C.E. (2025). Innovation in Osteogenesis Activation: Role of Marine-Derived Materials in Bone Regeneration. Curr. Issues Mol. Biol..

[13] Canto J.D., Mourão C.F., Moraschini V., da Silva Bonato R., Sartoretto S.C., Calasans-Maia M.D., Granjeiro J.M., Louro R.S. (2025). Teriparatide for Guided Bone Regeneration in Craniomaxillofacial Defects: A Systematic Review of Preclinical Studies. Curr. Issues Mol. Biol..

